# Supplementary material for: Coupling metabolic addiction with negative autoregulation to improve strain stability and pathway yield
Source: Metab Eng. 2020 Sep;61:79–88. doi: 10.1016/j.ymben.2020.05.005 (PMC7510839; doi:10.1016/j.ymben.2020.05.005)
Supplement: Multimedia component 1 [file mmc1.pdf]

Supplementary Materials

Coupling metabolic addiction with negative autoregulation to improve strain stability and pathway yield

Yongkun Lv [1,2,3], Yang Gu[1,3], Jingliang Xu[2], Jingwen Zhou[3] and Peng Xu [1*]

[1] Department of Chemical, Biochemical and Environmental Engineering, University of Maryland Baltimore County, Baltimore, MD 21250, USA.

[2] School of Chemical Engineering, Zhengzhou University, Zhengzhou, Henan 450001, China.

[3] National Engineering Laboratory for Cereal Fermentation Technology, Jiangnan University, Wuxi, Jiangsu 214122, China.

[*]Corresponding author Tel: +1(410)-455-2474; fax: +1(410)-455-1049. E-mail address: zhoujw1982@jiangnan.edu.cn (JWZ), and pengxu@umbc.edu (PX).

Supplementary figures

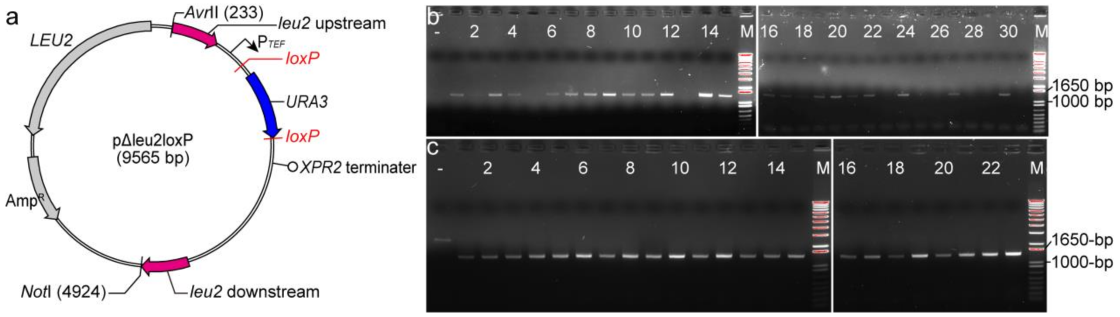

Supplementary Fig. 1 Site-directed integration at *leu2* loci and *URA3* marker curation. **a** Structure of plasmid pΔleu2loxP. *leu2* upstream and *leu2* downstream refer to the up and down homologous sequence of *leu2* loci. The *loxP* sites were used for *URA3* marker curation. **b** Colony PCR analysis of site-directed integration at *leu2* site. The colony PCR was carried out using primer pair leu2_Inte F/leu2_Inte R. The positive colony will yield a 1368-bp band, while the negative colony will not yield any specific band. **c** Colony PCR analysis of *URA3* marker curation. The colony PCR was carried out using primer pair 26srDNA2s F/XPR2_Seq. The positive colony will yield a 1533-bp band, while the negative colony will yield a 2432-bp band.

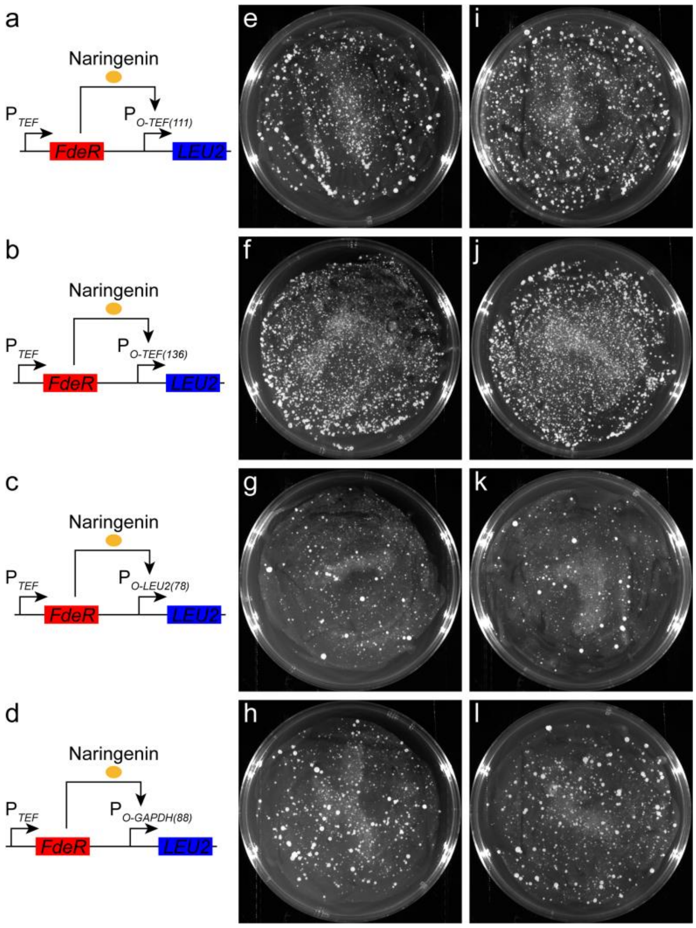

Supplementary Fig. 2 Analysis of naringenin addiction circuits on CSM-Leu and CSM-Leu+Naringenin plates. **a-d** Components of naringenin addiction circuits containing promoters $P_{O\text{-}TEF(111)}$, $P_{O\text{-}TEF(136)}$, $P_{O\text{-}LEU2(78)}$, and $P_{O\text{-}GAPDH(88)}$. **e-h** The control set. Growth of *Y. lipolytica* transformants containing promoters $P_{O\text{-}TEF(111)}$, $P_{O\text{-}TEF(136)}$, $P_{O\text{-}LEU2(78)}$, and $P_{O\text{-}GAPDH(88)}$ on CSM-Leu plates. Pictures were taken 3 days after transformation. **i-l** Growth of *Y. lipolytica* transformants containing promoters $P_{O\text{-}TEF(111)}$, $P_{O\text{-}TEF(136)}$, $P_{O\text{-}LEU2(78)}$, and $P_{O\text{-}GAPDH(88)}$ on CSM-Leu+Naringenin plates. Pictures were taken 3 days after transformation.

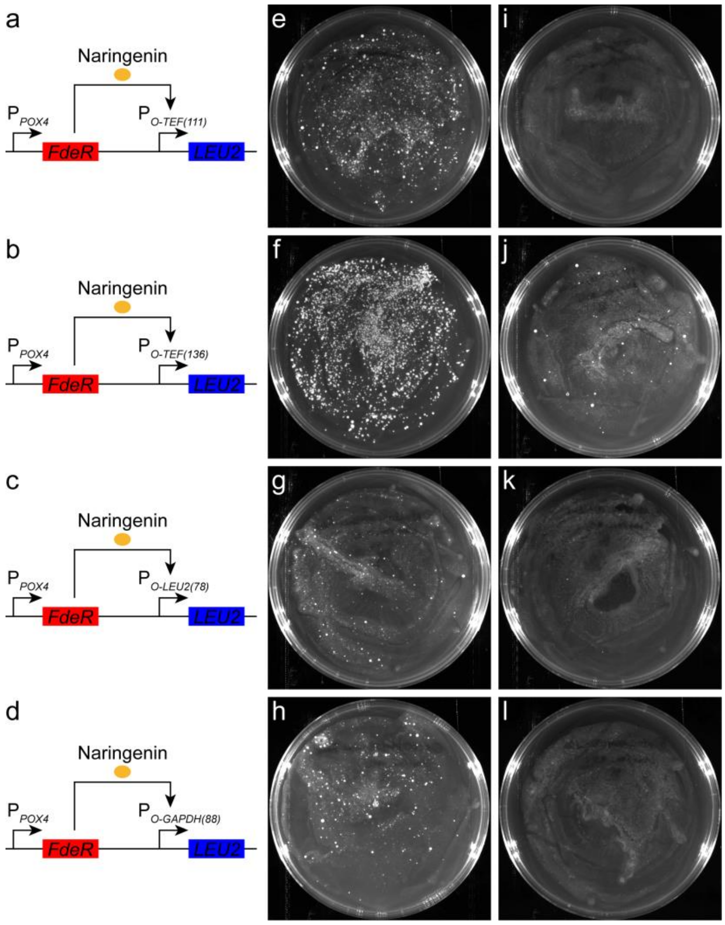

Supplementary Fig. 3 Analysis of complete and incomplete naringenin addiction circuits in naringenin producing strain NarPro/ASC_Rep. **a-d** Components of naringenin addiction circuits containing promoters $P_{O\text{-}TEF(111)}$, $P_{O\text{-}TEF(136)}$, $P_{O\text{-}LEU2(78)}$, and $P_{O\text{-}GAPDH(88)}$. FdeR was expressed using weaker promoter P_{POX4}. **e-h** Growth of NarPro/ASC_Rep containing complete addiction circuits on leucine drop-out CSM-leu plates. *LEU2* expression was controlled by $P_{O\text{-}TEF(111)}$, $P_{O\text{-}TEF(136)}$, $P_{O\text{-}LEU2(78)}$, and $P_{O\text{-}GAPDH(88)}$, respectively. Pictures were taken 3 days after transformation. **i-l** Negative control set. Growth of NarPro/ASC_Rep containing incomplete addiction circuits without FdeR on leucine drop-out CSM-leu plates. *LEU2* expression was controlled by $P_{O\text{-}TEF(111)}$, $P_{O\text{-}TEF(136)}$, $P_{O\text{-}LEU2(78)}$, and $P_{O\text{-}GAPDH(88)}$, respectively. Pictures were taken 3 days

after transformation.

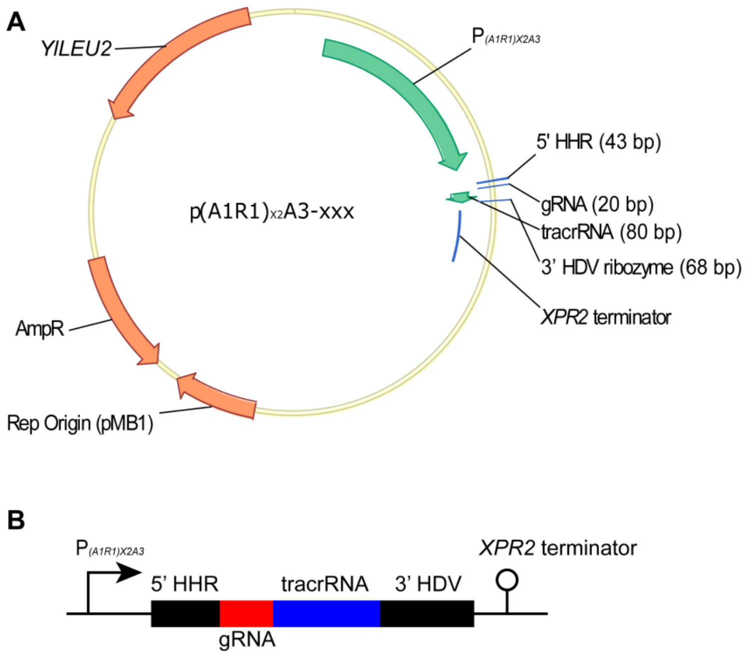

Supplementary Fig. 4. Genetic structure of plasmid p(A1R1)$_{x2}$A3-xxx. "xxx" here refers to gRNA. For instance, p(A1R1)$_{x2}$A3-FAS1-2 refers to p(A1R1)$_{x2}$A3 containing gRNA FAS1-2 generating components. **A.** Overall structure of plasmid p(A1R1)$_{x2}$A3-xxx. **B.** Structure of the gRNA generating components. The 5' HHR and 3' HDV ribozyme sites were used for generating mature gRNAs.

Supplementary tables

Supplementary Table 1 gRNA sequences and targeting locations

gRNA	Sequence (5'-3')	Targeting locations
FAS1-1	ACTCACCAAATCAAATAATG	UTR
FAS1-2	TTTTGCTACAGGAAACAGCG	Intron
FAS1-3	GGTGCAGTTGATGTACAGAG	CDS
FAS2-1	AAACCTGAGCCACAAATCAG	Intron
FAS2-2	GGGCATCACCACCAGCAGCG	CDS
FAS2-3	GAAGGGGACAACAATCAGAG	CDS
FabD-1	GCAGCATTCTTCCCCGGACA	CDS
FabD-2	GAGGCGTTGGATACCACCTG	CDS
FabD-3	CACATGATATACACAAAGCA	Intron

Supplementary Table 2 Plasmids used in this paper

Plasmid	Annotation
pYLXP'	A frozen stock of our Lab[1].
pYLXP'-Nluc	A frozen stock of our Lab[2].
pYLXP'-dCas9	A frozen stock of our Lab.
pYLXP'-Cre	A frozen stock of our Lab[3].
pYLXP'-URA3-loxP	A frozen stock of our Lab[3].
pΔleu2loxP	Integration at *leu2* site.
pΔxpr2loxP	Integration at *xpr2* site.
pΔleu2loxP-POX2(1591)-Nluc	Integrating POX2(1591)-Nluc at *leu2* site.
pΔleu2loxP-A1R1A3-Nluc	Integrating A1R1A3-Nluc at *leu2* site.
pΔleu2loxP-$(A1R1)_{x2}$A3-Nluc	Integrating $(A1R1)_{x2}$A3-Nluc at *leu2* site.
pΔleu2loxP-FAS1-1-dCas9	Integrating gRNA FAS1-1 and dCas9 circuits at *leu2* site.
pΔleu2loxP-FAS1-2-dCas9	Integrating gRNA FAS1-2 and dCas9 circuits at *leu2* site.
pΔleu2loxP-FAS1-3-dCas9	Integrating gRNA FAS1-3 and dCas9 circuits at *leu2* site.
pΔleu2loxP-FAS2-1-dCas9	Integrating gRNA FAS2-1 and dCas9 circuits at *leu2* site.
pΔleu2loxP-FAS2-2-dCas9	Integrating gRNA FAS2-2 and dCas9 circuits at *leu2* site.
pΔleu2loxP-FAS2-3-dCas9	Integrating gRNA FAS2-3 and dCas9 circuits at *leu2* site.
pΔleu2loxP-FabD-1-dCas9	Integrating gRNA FabD-1 and dCas9 circuits at *leu2* site.
pΔleu2loxP-FabD-2-dCas9	Integrating gRNA FabD-2 and dCas9 circuits at *leu2* site.
pΔleu2loxP-FabD-3-dCas9	Integrating gRNA FabD-3 and dCas9 circuits at *leu2* site.
pΔleu2loxP-FAS1-2-FAS2-1-dCas9	Integrating gRNAs FAS1-2 and FAS2-1 and dCas9 circuits at *leu2* site.
pΔleu2loxP-FAS1-2-FabD-1-dCas9	Integrating gRNAs FAS1-2 and FabD-1 and dCas9 circuits at *leu2* site.
pΔleu2loxP-FAS2-1-FabD-1-dCas9	Integrating gRNAs FAS2-1 and FabD-1 and dCas9 circuits at *leu2* site.
pΔleu2loxP-FAS1-2-FAS2-1-FabD-1-dCas9	Integrating gRNAs FAS1-2, FAS2-1, and FabD-1 and dCas9 circuits at *leu2* site.
pOTEF(111)	Replacing *TEF* promoter in pYLXP'2 with *OTEF(111)* hybrid promoter.
pOTEF(136)	Replacing *TEF* promoter in pYLXP'2 with *OTEF(136)* hybrid promoter.
pOLEU2(78)	Replacing *TEF* promoter in pYLXP'2 with *OLEU2(78)* hybrid promoter.
pOGAPDH(88)	Replacing *TEF* promoter in pYLXP'2 with *OGAPDH(88)* hybrid promoter.
pOTEF(111)-Nluc	Placing *Nluc* under the control of hybrid promoter *OTEF(111)*.
pOTEF(136)-Nluc	Placing *Nluc* under the control of hybrid promoter *OTEF(136)*.

pOLEU2(78)-Nluc	Placing *Nluc* under the control of hybrid promoter *OLEU2(78)*.
pOGAPDH(88)-Nluc	Placing *Nluc* under the control of hybrid promoter *OGAPDH(88)*.
pOTEF(111)-LEU2	Placing *LEU2* under the control of hybrid promoter *OTEF(111)*.
pOTEF(136)-LEU2	Placing *LEU2* under the control of hybrid promoter *OTEF(136)*.
pOLEU2(78)-LEU2	Placing *LEU2* under the control of hybrid promoter *OLEU2(78)*.
pOGAPDH(88)-LEU2	Placing *LEU2* under the control of hybrid promoter *OGAPDH(88)*.
pYLXP'-FdeR	Placing FdeR under the control of promoter *TEF*.
pYaliJ1-FdeR	Placing FdeR under the control of promoter *POX4*.
pYaliL1-FdeR	Placing FdeR under the control of promoter *IDP2*.
pOTEF(111)-FdeR	Placing *FdeR* under the control of hybrid promoter *OTEF(111)*.
pOTEF(136)-FdeR	Placing *FdeR* under the control of hybrid promoter *OTEF(136)*.
pOLEU2(78)-FdeR	Placing *FdeR* under the control of hybrid promoter *OLEU2(78)*.
pOGAPDH(88)-FdeR	Placing *FdeR* under the control of hybrid promoter *OGAPDH(88)*.
pOTEF(111)-Nluc-FdeR	pOTEF(111)-Nluc containing *FdeR* under the control of *TEF* promoter.
pOTEF(136)-Nluc-FdeR	pOTEF(136)-Nluc containing *FdeR* under the control of *TEF* promoter.
pOLEU2(78)-Nluc-FdeR	pOLEU2(78)-Nluc containing *FdeR* under the control of *TEF* promoter.
pOGAPDH(88)-Nluc-FdeR	pOGAPDH(88)-Nluc containing *FdeR* under the control of *TEF* promoter.
pOTEF(111)-LEU2-FdeR	pOTEF(111)-LEU2 containing *FdeR* under the control of *TEF* promoter.
pOTEF(136)-LEU2-FdeR	pOTEF(136)-LEU2 containing *FdeR* under the control of *TEF* promoter.
pOLEU2(78)-LEU2-FdeR	pOLEU2(78)-LEU2 containing *FdeR* under the control of *TEF* promoter.
pOGAPDH(88)-LEU2-FdeR	pOGAPDH(88)-LEU2 containing *FdeR* under the control of *TEF* promoter.
pOTEF(111)-LEU2-POX4-FdeR	pOTEF(111)-LEU2 containing *FdeR* under the control of *POX4* promoter.
pOTEF(136)-LEU2-POX4-FdeR	pOTEF(136)-LEU2 containing *FdeR* under the control of *POX4* promoter.
pOLEU2(78)-LEU2-POX4-FdeR	pOLEU2(78)-LEU2 containing *FdeR* under the control of *POX4* promoter.
pOGAPDH(88)-LEU2-POX4-FdeR	pOGAPDH(88)-LEU2 containing *FdeR* under the control of *POX4* promoter.
pOTEF(111)-LEU2-IDP2-FdeR	pOTEF(111)-LEU2 containing *FdeR* under the control of *IDP2* promoter.
pOTEF(136)-LEU2-IDP2-FdeR	pOTEF(136)-LEU2 containing *FdeR* under the control of *IDP2*

	promoter.
pOLEU2(78)-LEU2-IDP2-FdeR	pOLEU2(78)-LEU2 containing *FdeR* under the control of *IDP2* promoter.
pOGAPDH(88)-LEU2-IDP2-FdeR	pOGAPDH(88)-LEU2 containing *FdeR* under the control of *IDP2* promoter.
pOTEF(136)-LEU2-OTEF(111)-FdeR	pOTEF(136)-LEU2 containing *FdeR* under the control of *OTEF(111)* promoter.
pOTEF(136)-LEU2-OTEF(136)-FdeR	pOTEF(136)-LEU2 containing *FdeR* under the control of *OTEF(136)* promoter.
pOTEF(136)-LEU2-OLEU2(78)-FdeR	pOTEF(136)-LEU2 containing *FdeR* under the control of *OLEU2(78)* promoter.
pOTEF(136)-LEU2-OGAPDH(88)-FdeR	pOTEF(136)-LEU2 containing *FdeR* under the control of *OGAPDH(88)* promoter.
pΔxpr2loxP-OTEF(111)-Nluc-FdeR	Integrating OTEF(111)-Nluc-FdeR at *xpr2* site. Used for testing *OTEF(111)* promoter.
pΔxpr2loxP-OTEF(136)-Nluc-FdeR	Integrating OTEF(136)-Nluc-FdeR at *xpr2* site. Used for testing *OTEF(136)* promoter.
pΔxpr2loxP-OLEU2(78)-Nluc-FdeR	Integrating OLEU2(78)-Nluc-FdeR at *xpr2* site. Used for testing *OLEU2(78)* promoter.
pΔxpr2loxP-OGAPDH(88)-Nluc-FdeR	Integrating OGAPDH(88)-Nluc-FdeR at *xpr2* site. Used for testing *OGAPDH(88)* promoter.
pΔxpr2loxP-OTEF(111)-LEU2	Integrating OTEF(111)-LEU2 at *xpr2* site. Used as control.
pΔxpr2loxP-OTEF(136)-LEU2	Integrating OTEF(136)-LEU2 at *xpr2* site. Used as control.
pΔxpr2loxP-OLEU2(78)-LEU2	Integrating OLEU2(78)-LEU2 at *xpr2* site. Used as control.
pΔxpr2loxP-OGAPDH(88)-LEU2	Integrating OGAPDH(88)-LEU2 at *xpr2* site. Used as control.
pΔxpr2loxP-OTEF(111)-LEU2-FdeR	Integrating OTEF(111)-LEU2-FdeR at *xpr2* site.
pΔxpr2loxP-OTEF(136)-LEU2-FdeR	Integrating OTEF(136)-LEU2-FdeR at *xpr2* site.
pΔxpr2loxP-OLEU2(78)-LEU2-FdeR	Integrating OLEU2(78)-LEU2-FdeR at *xpr2* site.
pΔxpr2loxP-OGAPDH(88)-LEU2-FdeR	Integrating OGAPDH(88)-LEU2-FdeR at *xpr2* site.
pΔxpr2loxP-OTEF(111)-LEU2-POX4-FdeR	Integrating OTEF(111)-LEU2-POX4-FdeR at *xpr2* site.
pΔxpr2loxP-OTEF(136)-LEU2-POX4-FdeR	Integrating OTEF(136)-LEU2-POX4-FdeR at *xpr2* site.

pΔxpr2loxP-OLEU2(78)-LEU2-POX4-FdeR	Integrating OLEU2(78)-LEU2-POX4-FdeR at *xpr2* site.
pΔxpr2loxP-OGAPDH(88)-LEU2-POX4-FdeR	Integrating OGAPDH(88)-LEU2-POX4-FdeR at *xpr2* site.
pΔxpr2loxP-OTEF(111)-LEU2-IDP2-FdeR	Integrating OTEF(111)-LEU2-IDP2-FdeR at *xpr2* site.
pΔxpr2loxP-OTEF(136)-LEU2-IDP2-FdeR	Integrating OTEF(136)-LEU2-IDP2-FdeR at *xpr2* site.
pΔxpr2loxP-OLEU2(78)-LEU2-IDP2-FdeR	Integrating OLEU2(78)-LEU2-IDP2-FdeR at *xpr2* site.
pΔxpr2loxP-OGAPDH(88)-LEU2-IDP2-FdeR	Integrating OGAPDH(88)-LEU2-IDP2-FdeR at *xpr2* site.
pΔxpr2loxP-OTEF(136)-LEU2-OTEF(111)-FdeR	Integrating OTEF(136)-LEU2-OTEF(111)-FdeR at *xpr2* site.
pΔxpr2loxP-OTEF(136)-LEU2-OTEF(136)-FdeR	Integrating OTEF(136)-LEU2-OTEF(136)-FdeR at *xpr2* site.
pΔxpr2loxP-OTEF(136)-LEU2-OLEU2(78)-FdeR	Integrating OTEF(136)-LEU2-OLEU2(78)-FdeR at *xpr2* site.
pΔxpr2loxP-OTEF(136)-LEU2-OGAPDH(88)-FdeR	Integrating OTEF(136)-LEU2-OGAPDH(88)-FdeR at *xpr2* site.

Supplementary Table 3 Primers used in this paper

Primer	Sequence (5'-3')
leu2-up F	ATCCCTAAATTTGATGAAAGCCTAGGGCATAAAATGTGGAGAAGAAATC
leu2-up R	CCAACCCGGTCTCTGTCGTCTGTGGATGTGTGTGGTTGTATG
leu2-down F	AGCTTTACCGCAGCAGATCCGTCGTTTCTACGACGCATTGATGG
leu2-down R	CACTATTGGCCTATGCGGCCGCTGGCACTGAGCTCGTCTAACGG
xpr2-up F	ATCCCTAAATTTGATGAAAGCCTAGGGACGAGGACTCGTCCAACGG
xpr2-up R	CCAACCCGGTCTCTGTCGTCACCGAGAAGATCAGCGTTCTGTG
xpr2-down F	AGCTTTACCGCAGCAGATCCGACGCCAACACCAAGCTGGT
xpr2-down R	CACTATTGGCCTATGCGGCCGCGGACTCAGTAATAAGAGCCTCG
Nluc F	ACCAGCACTTTTTGCAGTACTAACCGCAGGTCTTCACACTCGAAGATTTCG
Nluc R	CATAGCACGCGTGTAGATACTTACGCCAGAATGCGTTCGCAC
LEU2 F	ACCAGCACTTTTTGCAGTACTAACCGCAGGAACCCGAAACTAAGAAGACCAAGA
LEU2 R	CATAGCACGCGTGTAGATACTTATACACTAGCGGACCCTGCCGGT
pPOX2(1591) F	CTAAATTTGATGAAAGCCTAGGGACGACGATATTCCGGTCCCGAAACCC
pPOX2(1591) R	CTGCCTCTGAAACTCACCATTCTAGAGGCGTCGTTGCTTGTGTG
A3 F	CGAATGGTACGATTCCGCCACAATTGGACATGTTTGTTTTTCCGATC
A1R1 R	GATCGGAAAAACAAACATGTCCAATTGTGGCGGAATCGTACCATTCG
A1R1_R1	GGTTTCGGGACCGGAATATCTGGCGGAATCGTACCATTCGC
A1R1_F2	GATATTCCGGTCCCGAAACCCC
A1R1_R2	TGGCGGAATCGTACCATTCGCC
gRNA F	TCCCTAAATTTGATGAAAGCCTAGGGACGACGATATTCCGGTCCC
gRNA R	CCTTTTATCAGACATAGTCGACTCCTCCGTTATTGTCTCGCTAGC
gRNA_FAS1-1 F	GACGAAACGAGTAAGCTCGTCACTCACCAAATCAAATAATGGTTTTAGAGCTAGAAATAG
gRNA_FAS1-1 R	ACGAGCTTACTCGTTTCGTCCTCACGGACTCATCAGACTCACCTGCGGTTAGTACTGCAA
gRNA_FAS1-2 F	GACGAAACGAGTAAGCTCGTCTTTTGCTACAGGAAACAGCGGTTTTAGAGCTAGAAATAG
gRNA_FAS1-2 R	ACGAGCTTACTCGTTTCGTCCTCACGGACTCATCAGTTTTGCCTGCGGTTAGTACTGCAA
gRNA_FAS1-3 F	GACGAAACGAGTAAGCTCGTCGGTGCAGTTGATGTACAGAGGTTTTAGAGCTAGAAATAG
gRNA_FAS1-3 R	ACGAGCTTACTCGTTTCGTCCTCACGGACTCATCAGGGTGCACTGCGGTTAGTACTGCAA
gRNA_FAS2-1 F	GACGAAACGAGTAAGCTCGTCAAACCTGAGCCACAAATCAGGTTTTAGAGCTAGAAATAG
gRNA_FAS2-1 R	ACGAGCTTACTCGTTTCGTCCTCACGGACTCATCAGAAACCTCTGCGGTTAGTACTGCAA
gRNA_FAS2-2 F	GACGAAACGAGTAAGCTCGTCGGGCATCACCACCAGCAGCGGTTTTAGAGCTAGAAATAG
gRNA_FAS2-2 R	ACGAGCTTACTCGTTTCGTCCTCACGGACTCATCAGGGGCATCTGCGGTTAGTACTGCAA

gRNA_FAS2-3 F	GACGAAACGAGTAAGCTCGTCGAAGGGGACAACAATCAGAGGTTTTAGAGCTAGAAATAG
gRNA_FAS2-3 R	ACGAGCTTACTCGTTTCGTCCTCACGGACTCATCAGGAAGGGCTGCGGTTAGTACTGCAA
gRNA_FabD-1 F	GACGAAACGAGTAAGCTCGTCGCAGCATTCTTCCCCGGACAGTTTTAGAGCTAGAAATAG
gRNA_FabD-1 R	ACGAGCTTACTCGTTTCGTCCTCACGGACTCATCAGGCAGCACTGCGGTTAGTACTGCAA
gRNA_FabD-2 F	GACGAAACGAGTAAGCTCGTCGAGGCGTTGGATACCACCTGGTTTTAGAGCTAGAAATAG
gRNA_FabD-2 R	ACGAGCTTACTCGTTTCGTCCTCACGGACTCATCAGGAGGCGCTGCGGTTAGTACTGCAA
gRNA_FabD-3 F	GACGAAACGAGTAAGCTCGTCCACATGATATACACAAAGCAGTTTTAGAGCTAGAAATAG
gRNA_FabD-3 R	ACGAGCTTACTCGTTTCGTCCTCACGGACTCATCAGCACATGCTGCGGTTAGTACTGCAA
TEF_F2	GGGTATAAAAGACCACCGTCCCC
26srDNA2s F	ATCCCTAAATTTGATGAAAGCCTAGGCAGACACTGCGTCGCTCCGTCC
XPR2_Seq	GGTGTTGGACTCAGTAATAAGAGCC
leu2_Inte F	GTGTGCACTCCAACTTTTCACAC
xpr2_Inte F	AGCCGTGTTTCGTGACGCAATC
leu2_Inte R	GATCATGCACACATAAGGTCC
$FAS1_{rt}$ F[1]	GTCTCTGTATGGTCTGTGTCTTG
$FAS1_{rt}$ R	GAGTGGAAAGGAGAGGTGATG
$FAS2_{rt}$ F	CACTCTCCCTTTTCTCCACATC
$FAS2_{rt}$ R	AGCAACCTCAAGACCATCG
$FabD_{rt}$ F	GCATCTCAAAGCCTCCAAAC
$FabD_{rt}$ R	TGAAATCGGGACGGATCTTG
$ACT1_{rt}$ F	GGTATCGTTCTTGACTCTGGTG
$ACT1_{rt}$ R	AGGTAGTCGGTAAGATCTCGG

1. The subscripts "$_{rt}$" refers to primers used for qRT-PCR.

References

1. Xu, P.; Qiao, K. J.; Ahn, W. S.; Stephanopoulos, G., Engineering *Yarrowia lipolytica* as a platform for synthesis of drop-in transportation fuels and oleochemicals. *Proc. Natl. Acad. Sci. U. S. A.* **2016,** *113* (39), 10848-10853.
2. Wong, L.; Engel, J.; Jin, E.; Holdridge, B.; Xu, P., YaliBricks, a versatile genetic toolkit for streamlined and rapid pathway engineering in *Yarrowia lipolytica*. *Metab. Eng. Commun.* **2017,** *5*, 68-77.
3. Lv, Y.; Edwards, H.; Zhou, J.; Xu, P., Combining 26s rDNA and the Cre-loxP system for iterative gene integration and efficient marker curation in *Yarrowia lipolytica*. *ACS Synth. Biol.* **2019**.
